# Hemolytic C-Type Lectin CEL-III from Sea Cucumber Expressed in Transgenic Mosquitoes Impairs Malaria Parasite Development

**DOI:** 10.1371/journal.ppat.0030192

**Published:** 2007-12-21

**Authors:** Shigeto Yoshida, Yohei Shimada, Daisuke Kondoh, Yoshiaki Kouzuma, Anil K Ghosh, Marcelo Jacobs-Lorena, Robert E Sinden

**Affiliations:** 1 Division of Medical Zoology, Department of Infection and Immunity, Jichi Medical University, Tochigi, Japan; 2 College of Agriculture, Ibaraki University, Ami-machi, Inashiki-gun, Ibaraki, Japan; 3 Department of Molecular Microbiology and Immunology, Malaria Research Institute, Johns Hopkins School of Public Health, Baltimore, Maryland, United States of America; 4 Infection and Immunity Section, Division of Cell and Molecular Biology, Imperial College London, South Kensington, London, United Kingdom; University of Minnesota, United States of America

## Abstract

The midgut environment of anopheline mosquitoes plays an important role in the development of the malaria parasite. Using genetic manipulation of anopheline mosquitoes to change the environment in the mosquito midgut may inhibit development of the malaria parasite, thus blocking malaria transmission. Here we generate transgenic Anopheles stephensi mosquitoes that express the C-type lectin CEL-III from the sea cucumber, Cucumaria echinata, in a midgut-specific manner. CEL-III has strong and rapid hemolytic activity toward human and rat erythrocytes in the presence of serum. Importantly, CEL-III binds to ookinetes, leading to strong inhibition of ookinete formation in vitro with an IC_50_ of 15 nM. Thus, CEL-III exhibits not only hemolytic activity but also cytotoxicity toward ookinetes. In these transgenic mosquitoes, sporogonic development of Plasmodium berghei is severely impaired*.* Moderate, but significant inhibition was found against Plasmodium falciparum. To our knowledge, this is the first demonstration of stably engineered anophelines that affect the *Plasmodium* transmission dynamics of human malaria. Although our laboratory-based research does not have immediate applications to block natural malaria transmission, these findings have significant implications for the generation of refractory mosquitoes to all species of human *Plasmodium* and elucidation of mosquito–parasite interactions.

## Author Summary

Malaria is arguably the most important vector-borne disease worldwide, affecting 300 million people and killing 1–2 million people every year. The lack of an effective vaccine and the emergence of the parasites' resistance to many existing anti-malarial drugs have aggravated the situation. Clearly, development of novel strategies for control of the disease is urgently needed. Mosquitoes are obligatory vectors for the disease and inhibition of parasite development in the mosquito has considerable promise as a new approach in the fight against malaria. Based on recent advances in the genetic engineering of mosquitoes, the concept of generating genetically modified (GM) mosquitoes that hinder transmission by either killing or interfering with parasite development is a potential means of controlling the disease. To generate these GM mosquitoes, the authors focused on a unique lectin isolated from the sea cucumber, which has both hemolytic and cytotoxic activities, as an anti-parasite effector molecule. A transgenic mosquito expressing the lectin effectively caused erythrocyte lysis in the midgut after ingestion of an infectious blood meal and severely impaired parasite development. This laboratory-acquired finding may provide significant implications for future malaria control using GM mosquitoes refractory to the parasites.

## Introduction

Malaria, transmitted by anopheline mosquitoes, is among the worst health problems in the world, killing 1–2 million people every year, mostly African children. Lack of an effective vaccine and the emergence of *Plasmodium* strains resistant to many existing anti-malarial drugs have aggravated this situation. Therefore, the control of vector competence has become a more important target in malaria intervention.

Recent advances in genetic engineering of anopheline mosquitoes have raised hopes for their use as new strategies for malaria control, also the provision of powerful tools for investigating mosquito-parasite interactions. We and others have characterized tissue-specific promoters that drive robust expression of transgenes in the midgut [1,2], hemocoel [3], and salivary glands [4]. The next challenge is to identify “effector” molecules to inhibit development of malaria parasites without competitive cost to the mosquito. To date, several effector molecules have been identified (e.g., single-chain antibody fragments directed against parasite ligands [5,6], the dodecapeptide SM1 [7], PLA2 [8], a cecropin-like peptide [9], and the Vida3 peptide [10]; (see reviews [11,12]). Of these, transgenic mosquitoes expressing either SM-1 or PLA2 in a midgut-specific manner were less able to support transmission of the rodent parasite P. berghei [13,14]. However, the SM1 transgenic mosquito was not resistant to the human malaria parasite P. falciparum (M. Jacobs-Lorena, unpublished observations), and the PLA2 transgenic mosquito was significantly less fit than the wild-type [15]. In those transgenic mosquitoes generated so far, no single effector molecule has exhibited a “non-sporozoite” phenotype in the salivary glands, i.e., complete *Plasmodium* transmission blockade. Therefore, other effector molecules and/or mechanisms are required to generate a transgenic mosquito that is both fit and refractory to all species and strains of human *Plasmodium*.

Transmission of malaria parasites is absolutely dependent on availability of competent mosquito vectors. Development of *Plasmodium* in the mosquito begins with ingestion of an infectious blood meal containing gametocytes from a vertebrate host [16]. In the mosquito midgut lumen, female and male gametocytes mature into gametes after exposure to environmental and mosquito-specific factors. These include a drop in temperature of 5 °C and exposure to xanthurenic acid [17]. A signal transduction cascade results in the release of calcium in the cytoplasm of the activated gametocyte, initiating development and its escape from the erythrocyte [18]. After fertilization, the zygote matures into a motile ookinete. Anopheline mosquitoes rapidly concentrate the contents of the blood meal 1.5- to 2-fold, resulting in highly viscous gut content. Although little is known about the influence of these changes, we postulated that changes to the midgut environment could inhibit parasite development. We chose to express the CEL-III lectin from the sea cucumber, Cucumaria echinata. CEL-III is a Ca^2+^-dependent (C-type) lectin, that exhibits strong hemolytic and cell-dependent activity [19] as well as cytotoxicity toward some cultured cell lines [20] by forming ion-permeable pores in target cell membranes through oligomerization after binding to carbohydrate chains on the cell surface [21,22]. Furthermore, synthetic peptides derived from the C-terminal hydrophobic region of CEL-III exhibit strong activity against Gram-positive bacteria such as Staphylococcus aureus and Bacillus subtilis [23].

Here we show that CEL-III strongly inhibits ookinete formation in vitro, and transgenic mosquitoes expressing CEL-III in the midgut significantly inhibit oocyst formation and sporozoite production, not only of P. berghei but also P. falciparum. To our knowledge, this is the first demonstration of stably engineered anophelines in which the reduction of vectorial capacity transcends *Plasmodium* species.

## Results

### Hemolytic and Hemagglutination Activities of CEL-III Are Directed Toward Human and Rat Erythrocytes, but Not Mouse Erythrocytes

**Figure 1:**
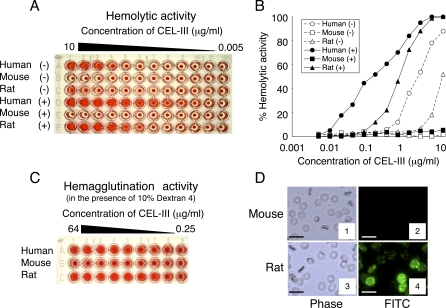
Hemolytic and Hemagglutination Activities of CEL-III toward Human, Mouse, and Rat Erythrocytes (A) Serial 2-fold dilutions of CEL-III were mixed with human, mouse, or rat erythrocytes in V-shaped microtiter plate wells. Samples were incubated in the absence (−) or presence (+) of 5% FBS. Hemolysis was examined visually after incubation for 1 h at room temperature. (B) Hemolytic activity toward human, mouse, and rat erythrocytes was expressed as the absorbance at 550 nm resulting from release of hemoglobin. (C) Serial 2-fold dilutions of CEL-III were mixed with human, mouse, or rat erythrocytes in V-shaped microtiter plate wells. Samples were incubated in the presence of 10% Dextran 4. Agglutination was examined visually after incubation for 1 h at room temperature. (D) CEL-III was added to mouse (panels 1 and 2) or rat erythrocytes (panels 3 and 4), and bound CEL-III was detected with FITC-labeled anti-mouse IgG following mouse anti-CEL-III antibody by fluorescence microscopy. Panels 1 and 3, phase contrast; panels 2 and 4, FITC. Scale bars are 10 μm.

CEL-III has strong Ca^2+^-dependent hemolytic activity toward human and rabbit erythrocytes, but shows only weak hemagglutination of chicken and horse erythrocytes [24]. This species-specific hemolysis is due to the binding of CEL-III to specific carbohydrate receptors on the erythrocyte surface. We examined whether CEL-III has hemolytic and hemagglutination activities toward mouse and rat erythrocytes as hosts for the rodent malaria parasite P. berghei. Figure 1A and 1B shows that the hemolytic activity of CEL-III was strong toward human and rat erythrocytes at low concentrations (IC_50_ = 0.3 and 0.8 μg/ml, respectively) in the presence of 5% fetal bovine serum (FBS: a source of Ca^2+^), whereas there was no hemolytic activity toward mouse erythrocytes. Weak hemolytic activity was observed against human and rat erythrocytes even in the absence of FBS. Similarly, CEL-III exhibited strong hemagglutination activity toward human and rat erythrocytes, but not toward mouse erythrocytes (Figure 1C). Fluorescent microscopic studies also confirmed that CEL-III bound to rat erythrocytes with numerous punctuate dots distributed throughout the cells, whereas no signals were detected in mouse erythrocytes (Figure 1D). These results suggest that carbohydrate chains on the mouse and rat erythrocyte surface may differ.

### In Vitro Effect of CEL-III on Ookinete Development

**Figure 2:**
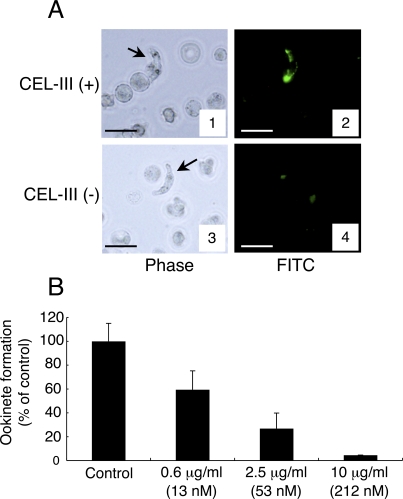
Binding of CEL-III to Ookinetes Inhibits Parasite Development In Vitro (A) Binding of CEL-III to ookinetes. CEL-III was added to cultured ookinetes purified from P. berghei–infected mouse blood. Bound CEL-III was detected with FITC-labeled anti-mouse IgG following mouse anti-CEL-III antibody by fluorescence microscopy (panels 1 and 2) (CEL-III (+)). As a negative control, ookinetes were incubated with FITC-labeled anti-mouse IgG following mouse anti-CEL-III antibody without CEL-III (panels 3 and 4) (CEL-III (−)). Panels 1 and 3, phase contrast; panels 2 and 4, FITC. Arrows indicate cultured ookinetes. Scale bars are 10 μm. (B) Effect of CEL-III on ookinete development in vitro. P. berghei–infected mouse blood was cultured for ookinetes for 24 h at 19 °C. CEL-III was added at initiation of the culture at various concentrations. Data are expressed as number of ookinetes relative to medium alone (100%). Results are the mean of two independent experiments, and bars represent standard errors of the mean.

It has been reported that CEL-III is cytotoxic toward some cultured cell lines as well as toward erythrocytes [20]. Therefore, we investigated the effect of CEL-III on ookinete development in vitro. At first, CEL-III was added to cultured ookinetes in the absence of Ca^2+^. Figure 2A shows that bound CEL-III was observed as small punctuate dots distributed throughout the ookinete (similar the binding of CEL-III to rat erythrocytes as shown in Figure 1D), whereas no signals were detected in the ookinete without CEL-III. Next, CEL-III was incubated with gametocytes in vitro and the number of ookinetes was determined 24 h later. Figure 2B shows that CEL-III (10 μg/ml) inhibited ookinete development by approximately 95%. This inhibition was dose-dependent, with an IC_50_ of approximately 15 nM.

### CEL-III Is Expressed in the Midgut of Transgenic Mosquitoes

**Figure 3:**
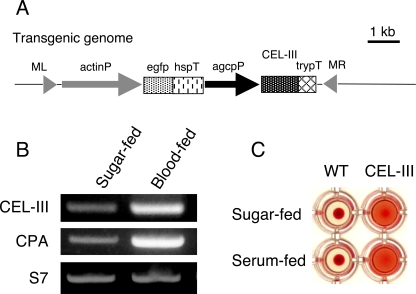
Structure of the *CEL-III* Gene and Its Expression in Transgenic Mosquitoes (A) Schematic diagram of the pMinos-EGFP-carboxypeptidaseP-CELIII-antryp1T construct used for A. stephensi germ line transformation. The construct consists of the D. melanogaster actin5c promoter (actinP), egfp selectable marker (egfp), and D. melanogaster hsp70 terminator sequence (hspT), the A. gambiae carboxypeptidase promoter sequence (agcpP) plus its signal sequence (SP), fused in-frame to the coding sequence of CEL-III without its signal sequence followed by the A. gambiae trypsin terminator sequence (trypT). The left (ML) and right (MR) arms of Minos are indicated by triangles. (B) Induction of CEL-III mRNA by a blood meal. Transgenic mosquitoes were allowed to feed on a non-infected mouse and 6 h later total RNA was extracted from midguts of engorged mosquitoes (Blood-fed). As a control, total RNA was extracted from midguts of sugar-fed mosquitoes (Sugar-fed). CEL-III mRNA level was examined using RT-PCR. PCR products of the endogenous carboxypeptidase gene and the S7 gene were used as inducible positive controls and quantitative controls of the different mRNA preparations, respectively. These PCR products were fractionated by electrophoresis then stained with ethidium bromide. (C) Hemolytic activity of midgut contents of transgenic mosquitoes. Transgenic (CEL-III) and non-transgenic (WT) mosquitoes were offered a serum meal by membrane feeding. Six h after the meal, the supernatants of midgut lysates were added to human erythrocytes. Hemolytic activity was determined by visual examination of lysis of erythrocytes as described in Figure 1A.

To express CEL-III in the A. stephensi midgut, we made a *pAgCP-CEL-III* gene cassette consisting of the promoter, 5′-UTR, and signal peptide from the A. gambiae carboxypeptidase A (*AgCPA*) gene [1] linked to the coding sequence of the *CEL-III* gene that lacked signal peptide sequence and the anopheles trypsin 1 (*Antryp1*) putative terminator region (Figure 3A). This gene cassette was inserted into pMinos-EGFP-RfA-F to construct pMinos-EGFP-carboxypeptidaseP-CELIII-antryp1T, then transformed into the germ line of A. stephensi embryos. A total of 876 embryos were injected and 22 fertile G_0_ matings were obtained. From these, one mating produced transgenic offspring expressing the *egfp* selectable marker. A transgenic homozygous line was obtained and propagated. A single integration event was confirmed by Southern blot analysis using genomic DNA from G_4_ adults (data not shown). The transgenic line has been stably maintained by blood feeding on mice or rats for over 30 generations, with no difference in reproductive fitness between transgenic and non-transgenic mosquitoes (i.e., number of eggs and hatched larvae; data not shown).

Expression profiles of the *CEL-III* transgene were investigated by real-time (RT)-PCR (Figure 3B). *CEL-III* mRNA was present in the midgut cells of sugar-fed mosquitoes and was strongly induced 6 h after blood ingestion, consistent with the pattern of expression of the endogenous A. stephensi carboxypeptidase A (*AsCPA*) gene, which is similar expression pattern to that of the *AgCPA* gene [25]. To examine whether CEL-III is secreted into the midgut lumen upon blood ingestion, transgenic mosquitoes were offered a serum meal by membrane feeding. Midgut lysates of the transgenic mosquitoes before and after the serum meal contained hemolytic activity toward human erythrocytes, but not in those of non-transgenic mosquitoes, indicating CEL-III is secreted into the midgut lumen upon feeding (Figure 3C).

**Figure 4:**
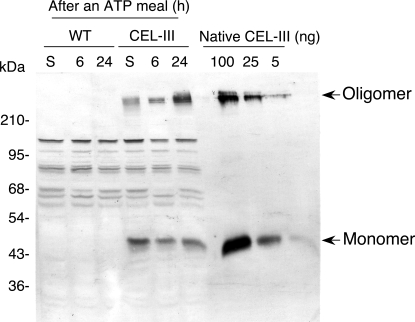
CEL-III Expression in Midguts of Transgenic Mosquitoes Transgenic (CEL-III) and non-transgenic (WT) mosquitoes were allowed to feed on naïve mice. After 6 or 24 h, midguts of engorged mosquitoes were dissected and lysed, then electrophoresed on 8% SDS-PAGE. As a control, midguts of sugar-fed mosquitoes (S) were dissected and lysed. CEL-III expression level was examined by western blotting using mouse anti-CEL-III antiserum. Each lane contains protein lysates equivalent to two midguts. The source of protein is indicated at the top of each lane (6, 24 h). For quantitative estimation of CEL-III per midgut, native CEL-III isolated from C. echinata body fluid was analyzed by western blotting. The amount of native CEL-III (5, 25, 100 ng) is indicated at the top of each lane. Arrows indicate the positions of monomeric and oligomeric forms of CEL-III.

Immunoblot analysis detected monomeric (48 kDa) and oligomeric (>200 kDa) forms of CEL-III in the midguts of sugar-fed transgenic mosquitoes (Figure 4). The relative mobilities of these two forms were similar to those of native CEL-III. In mosquitoes offered a blood-free ATP meal as a phagostimulant, the *AgCPA* promoter is activated [25,26]. Under these conditions, a slightly enhanced expression of the oligomeric form was observed 24 h after the meal. Compared to the native CEL-III, we estimate 5–10 ng of CEL-III accumulated in a single midgut after the ATP meal.

### Transgenic Mosquitoes Completely Hemolyze Human Erythrocytes 24 h after a Blood Meal

**Figure 5:**
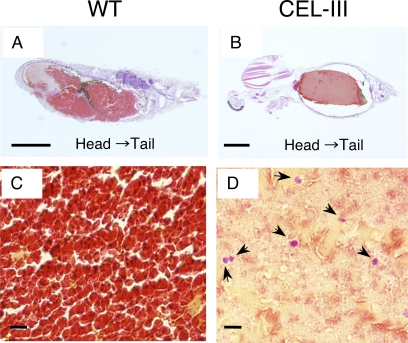
Hemolysis of Human Blood in Mosquito Midgut Mosquitoes were allowed to feed on a human volunteer. Representative photomicrographs of engorged mosquito gut sections 24 h after a blood meal are shown (HE staining, ×40 magnification for [A and B], and ×1,000 magnification for [C and D]). Midgut of non-transgenic mosquitoes was filled with intact erythrocytes (A and C), with many spaces between erythrocytes. In contrast, no space is observed in the midgut of transgenic mosquitoes (B and D). Erythrocytes appear to be completely hemolyzed, and HE-stained lymphocytes cells are detectable (arrows). Scale bars in (A and B) and (C and D) are 500 μm and 10 μm, respectively.

We confirmed hemolysis of human erythrocytes 24 h after a blood meal in midgut sections of a transgenic mosquito. Mosquitoes were allowed to feed on a human, then, 24 h after the blood feeding, gut sections were prepared for histology and stained with hematoxylin and eosin (HE). Compared to non-transgenic midguts which were filled with intact erythrocytes (Figure 5A and 5C), erythrocytes in the midgut of transgenic mosquito were extensively hemolyzed (Figure 5B and 5D). Lymphocytes were clearly contrasted in the midgut of transgenic mosquito (Figure 5D), but not amongst the intact erythrocytes in the midgut of non-transgenic mosquito (Figure 5C). These results are consistent with the data shown in Figure 3C, where the secretion of CEL-III into the midgut lumen caused effective hemolysis.

### Transgenic Mosquitoes Impair P. berghei Oocyst Formation in Both Rat and Mouse Models

To investigate the effect of CEL-III expression on P. berghei development, both transgenic and non-transgenic mosquitoes were allowed to feed on the same P. berghei–infected rat and the number of oocysts formed was counted. In three experiments, the infection rate (prevalence) of transgenic mosquitoes (10.5%) was markedly reduced compared to non-transgenic mosquitoes (63.6%) (transmission blockade of prevalence, TBp; 83.5%, *p* < 0.01). The oocyst numbers were consistently and strongly lower in transgenic mosquitoes (transmission blockade of intensity, TBi; range 90.0 to 97.9%, mean 90.5%, *p* < 0.01) (Table 1). For mouse experiments, TBp in Experiment 1 and TBi in both experiments were significantly reduced in transgenic mosquitoes. Overall, the two experiments combined, both TBp and TBi were significantly reduced in transgenic mosquitoes (*p* < 0.01) (Table 2).

**Table 1:**
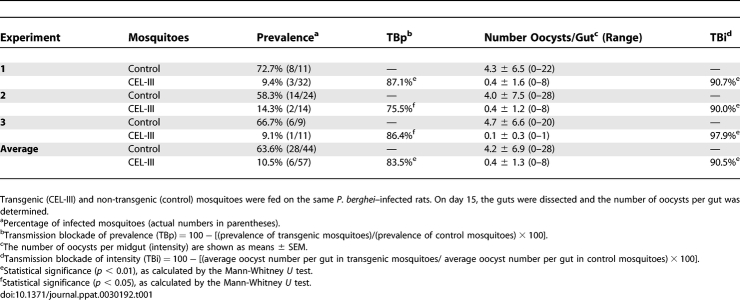
Rat–P. berghei Experiments

**Table 2:**
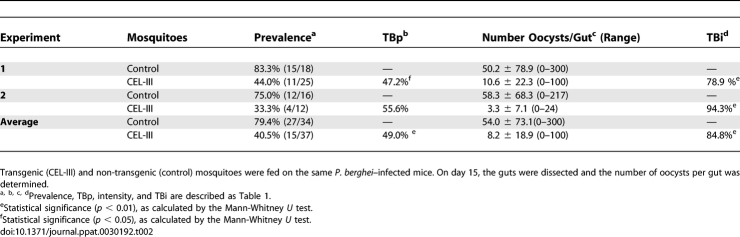
Mouse–P. berghei Experiments

### Vector Competence for P. berghei in Transgenic Mosquitoes Is Reduced

The impact of CEL-III expression on the ability of mosquitoes to transmit the parasite to uninfected animals (vectorial competence) was measured (Table 3). Vectorial competence of transgenic mosquitoes (20%) was severely impaired, compared to non-transgenic mosquitoes (100%). After a blood meal, the salivary glands of engorged mosquitoes were dissected, and numbers of sporozoites were counted. The number of sporozoites in individual salivary glands of the transgenic mosquitoes was markedly lower than that of non-transgenic mosquitoes. Importantly, sporozoite prevalence in transgenic mosquitoes (10%) was significantly reduced compared to non-transgenic mosquitoes (60%) (Table 4). These data reflect the oocyst prevalence seen in the rat experiments (Table 1).

**Table 3:**
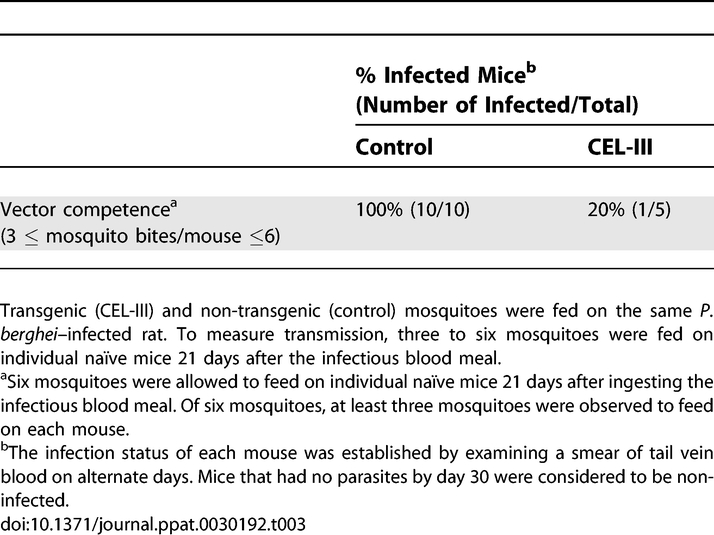
Vectorial Competence of Transgenic Mosquitoes

**Table 4:**
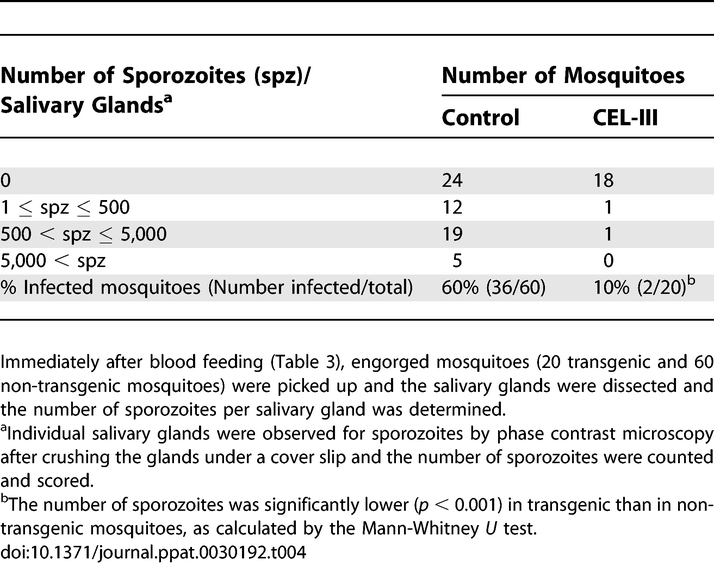
Sporozoite Infectivity of Salivary Glands of Transgenic Mosquitoes

### Transgenic Mosquitoes Impair Oocyst Formation of P. falciparum

To investigate the effect of CEL-III expression on human *Plasmodium* development, both transgenic and non-transgenic mosquitoes were allowed to feed on mature P. falciparum gametocyte cultures by membrane feeding, followed by determination of the number of oocysts formed (Table 5). In Experiment 1, oocyst formation in transgenic mosquitoes was significantly impaired (TBi 76.6%). In Experiment 2, TBi was 57.1%, and there was no statistically significant difference between transgenic and non-transgenic mosquitoes. Most likely, the low infection prevalence (30%) and low oocyst number (0.7 ± 1.4) in Experiment 2 affected the statistical analysis. Overall, with the two experiments combined, transgenic mosquitoes significantly impaired P. falciparum oocyst numbers (TBi 69.1%, *p* < 0.05), although TBp was only 7.8%.

**Table 5:**
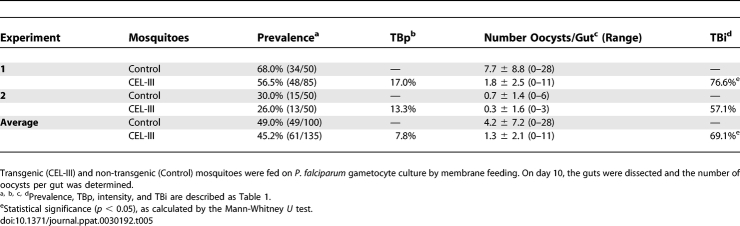
P. falciparum Gametocyte Membrane Feeding Experiments

## Discussion

This study demonstrates a novel “proof-of-concept” showing that transgenic mosquitoes expressing C-type lectin CEL-III significantly impairs development of both P. berghei and P. falciparum. We hypothezised that an environmental change in the midgut of anopheline mosquitoes by genetic manipulation could provide a new strategy for interrupting parasite development. CEL-III is a C-type, galactose/N-acetylgalactosamine (GalNAc)-specific lectin isolated from the body fluid of the marine invertebrate C. echinata. This lectin exhibits strong and rapid hemolytic activity and cytotoxicity through pore formation in target cell membranes. CEL-III is thought to play an important role in innate defense systems of C. echinata and therefore has the potential not only to change the environment of the mosquito midgut by rapid hemolysis of a blood meal, but also to act directly as a toxin against parasites.

For CEL-III, the N-terminal region contains two carbohydrate binding domains that have homology with the B-chains of ricin and abrin [20,27,28] and binds to the carbohydrate chains on the surface of the target cell membrane by its lectin activity. The C-terminal hydrophobic region that has antibacterial activity is believed to permeabilize the lipid bilayer of target microbes and cells [23]. CEL-III may therefore exhibit direct effector function to kill parasites. Alternatively, similar to other antimicrobial peptides or lectins, CEL-III may induce cells to undergo apoptosis [29].

In the transgenic mosquitoes, CEL-III is constitutively expressed prior to blood meal ingestion and accumulates in the midgut. Expression level of CEL-III was enhanced and reached a peak at 5–10 ng per midgut after a protein-free ATP meal. This amount is sufficient to completely hemolyze human erythrocytes in 3 μl-whole blood. As A. stephensi usually imbibes in less than 2.2 μl in a single blood meal [30], this result is consistent with the observation that complete hemolysis occurred in the midgut at 24 h after a blood meal.

Within minutes of ingestion, both male and female gametocytes escape from enveloping erythrocytes, then transform into male and female gametes. The male gametes produce eight flagellate microgametes in a process termed exflagellation, fertilizing the female gametes, giving rise first to zygotes then motile ookinetes. In the rat model, CEL-III accumulation in the midgut before a blood meal is likely to hemolyze erythrocytes infected with gametocytes immediately after a blood meal. As a result, extracellular gametocytes may be killed before differentiation. Although CEL-III cannot cause hemolysis of mouse erythrocytes, oocyst formation was also significantly reduced in the mouse model suggesting that direct parasite toxicity may be the dominant impact of the peptide. Preliminary observations suggest CEL-III reduces the efficiency of fertilization (S.Y. unpublished data). Additionally CEL-III bound to cultured ookinetes correlating with a strong killing effect on the parasites (IC_50_ = 15 nM) at 100- to 1,000-fold lower concentrations in vitro, when compared to other reported effector molecules, such as cecropin-like peptide [9], defensin [31], Vida3 [10], SM1 [7], and PLA2 [8]. In the rat model, the higher TBi (90.5%) may be due to additional hemolysis compared to that of the mouse model (84.8%). Although the binding specificity and mechanism by which CEL-III kills parasites in mosquitoes is unknown, findings from this study suggest that CEL-III may cause lethal damage to the female gamete and ookinete by pore formation following oligomer formation.

For P. berghei, the key property, as proposed in 1968 by Curtis [32], of vectorial competence was demonstrably and severely impaired, as measured by the relative inefficiency of transgenic mosquitoes to infect naïve mice compared to wild-type. Importantly, CEL-III transgenic mosquitoes impair sporogonic development of P. falciparum. To our knowledge, this is the first demonstration of stably engineered anophelines that affect the human *Plasmodium* transmission dynamics of a human malaria*.* Compared to the P. berghei-rat model, the TBi of P. falciparum is numerically lower (69.1%). One possible explanation for the lower TBi is that membrane feeding of in vitro cultured P. falciparum gametocytes does not contain leukocytes that may remain active in the mosquito blood meal and kill or phagocytose the liberated extracellular parasites [33,34].

In malaria endemic areas, multiple infections with *Plasmodium* species and strains are often observed. Those effector gene products must inhibit development of all species and strains of *Plasmodium* in the mosquito. As CEL-III targets erythrocytes, the “vehicles” for this parasite, as well as ookinetes, this transgenic mosquito may prove to be refractory to all species and strains of *Plasmodium*, including P. falciparum and P. vivax. Transgenic mosquitoes must have a minimal fitness cost, as such costs would reduce the effectiveness of the genetic drive mechanisms used to introduce transgenes into field mosquito populations. To date, there have been no single or cumulative toxic effects observed from CEL-III production in mosquitoes for fecundity (eggs laid per female). Further studies are nevertheless required to address the ability of CEL-III transgenic mosquitoes to compete with their non-transgenic siblings.

While we have demonstrated it is possible to create mosquitoes with impaired vectorial competence for more than one species of malarial parasites, we recognize there are numerous other scientific and ethical problems to be overcome before such a control strategy could be implemented.

## Materials and Methods

### Mosquitoes, animals, and parasites.

A. stephensi mosquito strain SDA 500 was maintained at Jichi Medical University and Imperial College London. Female BALB/c mice were obtained from SEASCO (Saitama, Tokyo, Japan) and used at 7 to 8 weeks of age. Female brown Norway rats were obtained from SEASCO and used at 7 to 8 weeks of age. P. berghei strain ANKA 234 was maintained by cyclical passage through Balb/c mice and A. stephensi using standard methods [35]. P. falciparum strain 3D7 was maintained in asynchronous culture as described elsewhere [36].

### Hemolytic and hemagglutination and assay.

CEL-III was purified from C. echinata body fluid as previously described [22]. Hemolytic activity was measured in the absence or presence of 5% FBS either by visual examination of lysis of erythrocytes or by measurement of hemoglobin release from erythrocytes using absorbance at 540 nm, as previously described [24]. Hemagglutination activity of CEL-III toward human, mouse, and rat erythrocytes was measured in the presence of 10% Dextran 4 (an osmotic protectant: SERVA, Heidelberg, Germany) as previously described [19].

### In vitro ookinete inhibition assay.

P. berghei–infected mouse blood was diluted in 5 vol ookinete medium (RPMI 1640, 10% FCS, 50 μg/ml hypoxanthine, 0.024 M NaHCO_3_, 5 μg/ml penicillin and 5 μg/ml streptomycin, final pH 8.3) in 24-well plates with different concentrations of CEL-III and control containing the same buffer. The plate was then incubated at 19 °C on a slow moving shaker for 24 h. After 24 h, the culture was then smeared and fixed with methanol. Air-dried slides were stained with Giemsa, and then the number of ookinetes was counted in a sample of 2,000 or 5,000 RBCs.

### Binding of CEL-III to erythrocytes and ookinetes.

Mouse and rat erythrocytes were prepared from whole bloods by washing five times with PBS. In vitro cultured ookinetes were purified as previously described [37]. Mouse erythrocytes, rat erythrocytes, or ookinetes were incubated with 25 μg/ml of CEL-III at room temperature for 1 h in PBS, and then washed five times with PBS. Bound CEL-III was detected by fluorescence microscopy with goat FITC-labeled anti-mouse IgG (Biosource) following mouse anti-CEL-III antiserum [19].

### Minos vector construction and germline transformation.

PCR reactions were performed with *Pfu* DNA polymerase (Stratagene GmbH). A gene fragment encoding amino acids 11–342 of CEL-III was amplified from plasmid pGEM-CEL-III [38] by PCR using primers pCEL-III-F1 and -R1 (Table S1). The PCR product was cloned into pENTR/D-TOPO (Invitrogen) to generate pENTR-CEL-III. A 2,311-bp DNA fragment of the putative promoter region of the *AgCPA* gene and its signal sequence was obtained from A. gambiae genomic DNA by PCR using primers pAgCPA-F2 and -R2 (Table S1). A 392-bp DNA fragment of the putative terminator region of *Antryp1* [39] was obtained from A. gambiae genomic DNA by PCR using primers pAgAntrp1-F1 and -R1 (Table S1). The *AgCPA* promoter and *Antryp1* terminator were assembled by overlapping PCR using primers pAgCPA-F2 and pAgAntrp1-R1, then cloned into pENTR/D-TOPO (Invitrogen) to generate pENTR-carboxypeptidaseP-antryp1T. The gene fragment encoding CEL-III was excised from pENTR-CEL-III by digestion with BglII and SphI, then cloned into the BamHI/SphI sites of pENTR-carboxypeptidaseP-antryp1T to generate plasmid pENTR-carboxypeptidaseP CEL-III-antryp1T. Transformation plasmid pMinos-EGFP-carboxypeptidaseP-CELIII-antryp1T was generated by incubation of pMinos-EGFP-RfA-F [4] and pENTR-carboxypeptidaseP-CELIII-antryp1T in the presence of LR Clonase (Invitrogen) according to the manufacturer's instructions. Primer sequence information is available in Table S1.

Embryo microinjection of the transformation and helper plasmids, screening of EGFP-expressing G_0_-G_2_ larvae, and generation of a homozygous line were performed as previously described [4].

### RT-PCR.

We have cloned and sequenced a gene fragment encoding a part of the *AsCPA* gene from the midgut mRNA of A. stephensi by RT-PCR using primers, pAgCPA-F1 and pAgCPA-R1 (Table S1), designed for the *AgCPA* gene. Total RNA was isolated from mosquito midguts using an RNeasy Mini column (Qiagen). Gene-specific primers for the *CEL-III*, *AsCPA*, and ribosomal protein *S7* genes were pCEL3-RT-F1/pCEL3-R2, pAsCPA-F1/pAsCPA-R1, and pAgS7-F1/pAgS7-R1, respectively (Table S1). Aliquots of cDNA representing 0.2 μg total RNA were amplified by PCR using the primer sets for detection of these genes. PCR products were separated by electrophoresis on a 2% agarose gel then visualized by ethidium bromide staining. PCR products of the *S7* gene were used as controls for quality of the different mRNA preparations used in the RT-PCR analysis.

### Hemolytic assay for mosquito midgut contents.

Mosquitoes were offered RPMI1640 medium containing 50% FBS through a Parafilm membrane warmed to 37 °C with a glass-watered jacket. Six h after the meal, engorged midguts from 5 mosquitoes were dissected in TBS-Ca (10 mM Tris-HCl [pH 7.5], 150 mM NaCl and 10 mM CaCl_2_), then homogenized in a small volume of TBS-Ca buffer, and supernatants were removed by centrifugation. The supernatants were added to human erythrocytes, and then hemolytic activity was measured by visual examination of lysis of erythrocytes as described above.

### Immunoblot analysis.

Mosquitoes were offered protein-free ATP solution (1 mM ATP, 150 mM NaCl, 10 mM NaHCO_3_ [pH 7.0]) through a Parafilm membrane warmed to 37 °C with a glass-watered jacket. This protein-free ATP solution was used to minimize background in subsequent western blots as previously described [25]. Engorged midguts were dissected 6 or 24 h after the meal in phosphate buffered saline (PBS), then solubilized with Laemmli buffer containing 1% 2-mercaptoethanol. The equivalent of 2 guts was separated on an 8% SDS-PAGE, electroblotted to Immobilon Transfer Membrane (Millipore), then probed with mouse anti-CEL-III polyclonal antibody. Bound antibodies were subsequently detected as previously described [5]. Native CEL-III was used for quantification of CEL-III expression per gut.

### Histology of midgut sections.

Mosquitoes were allowed to feed on a healthy Japanese volunteer. 24 h after a blood meal, engorged mosquitoes were fixed with 10% buffered formalin, then embedded in paraffin wax. Each block was cut into 4-μm sections, and then stained with HE. The Japanese volunteer gave his written consent to be included in this study after detailed explanation of the research project.

### Oocyst inhibition assay for *P. berghei.*

Transgenic and sibling non-transgenic mosquitoes were mixed in the same container then allowed to feed on a single infected rat or mouse. Blood-fed mosquitoes were separated after 24 h, then sorted into transgenic and non-transgenic mosquitoes using a fluorescence stereomicroscope SZX7 (Olympus) with GFP filter (excitation/emission at 480 nm/515 nm). Expression of EGFP in the abdomen of transgenic mosquitoes allowed them to be distinguished from non-transgenic mosquitoes. The two species of mosquitoes were separately housed in pots at 21 °C with 5% fructose solution. On day 15, midguts were dissected, then number of oocysts per midgut was determined. Prevalence, TBp, the mean number of oocysts in the midgut (intensity), and TBi were calculated as previously described [5,40]. Data were analyzed using the Mann-Whitney *U* test.

### Oocyst inhibition assay for *P. falciparum.*

Mature gametocytes of P. falciparum (3D7) were produced in vitro as previously described [41]. Membrane feeding assays were performed to test infectivity of the P. falciparum gametocytes for mosquitoes as previously described [42]. Briefly, mature gametocyte cultures (0.3 to 0.4% final gametocytemia) were fed for 30 min at 37 °C to transgenic and non-transgenic mosquitoes through a Parafilm membrane. Engorged mosquitoes were housed in pots at 26 °C and 60%–80% relative humidity. On day 10, midguts were dissected and number of oocysts per midgut was determined. The prevalence was analyzed as above.

### Sporozoite transmission assay.

Transgenic and non-transgenic mosquitoes were allowed to feed on the same rat, which was infected with P. berghei. Mosquitoes that blood-fed (30 transgenic and 60 non-transgenic mosquitoes) were separated after 24 h and housed in pots at 21°C with 5% fructose solution. To measure transmission, 6 mosquitoes per group were allowed to feed on individual naïve mice 21 days after ingesting the infectious blood meal. Of 6 mosquitoes, at least 3 mosquitoes were observed to feed on each mouse. Immediately after a blood meal, engorged mosquitoes (20 of 30 transgenic and all 60 non-transgenic mosquitoes) were picked up and the salivary glands were excised, placed on a microscope slide, squashed under a cover slip, and then examined by phase-contrast microscopy (× 400). Numbers of sporozoites per salivary gland (intensity) was determined using a gland index based on Collins et al*.* (1977) [43]: 0; 1: 1–499; 2; 500–4,999; 3: > 5,000. The infection status of each mouse was established by examining a smear of tail blood on alternate days. Mice that had no parasites by day 30 were considered to be uninfected.

## Supporting Information

Table S1 Primer Sequences (40 KB DOC)

### Accession Numbers

The GenBank (http://www.ncbi.nlm.nih.gov/Genbank/) accession numbers for the genes discussed in this paper are *AsCPA* (AB353072) and *CEL-III* (AB109017).

## Abbreviations

AsCPA: Anopheles stephensi carboxypeptidase A
AgCPA: Anopheles gambiae carboxypeptidase A
C-type: Ca2+-dependent
HE: hematoxylin and eosin
TBp: transmission blockade of prevalence
TBi: transmission blockade of intensity

## References

[ppat-0030192-b001] Moreira LA, Edwards MJ, Adhami F, Jasinskiene N, James AA (2000). Robust gut-specific gene expression in transgenic Aedes aegypti mosquitoes. Proc Natl Acad Sci U S A.

[ppat-0030192-b002] Abraham EG, Donnelly-Doman M, Fujioka H, Ghosh A, Moreira L (2005). Driving midgut-specific expression and secretion of a foreign protein in transgenic mosquitoes with AgAper1 regulatory elements. Insect Mol Biol.

[ppat-0030192-b003] Kokoza V, Ahmed A, Cho WL, Jasinskiene N, James AA (2000). Engineering blood meal-activated systemic immunity in the yellow fever mosquito, Aedes aegypti. Proc Natl Acad Sci U S A.

[ppat-0030192-b004] Yoshida S, Watanabe H (2006). Robust salivary gland-specific transgene expression in Anopheles stephensi mosquito. Insect Mol Biol.

[ppat-0030192-b005] Yoshida S, Matsuoka H, Luo E, Iwai K, Arai M (1999). A single-chain antibody fragment specific for the Plasmodium berghei ookinete protein Pbs21 confers transmission blockade in the mosquito midgut. Mol Biochem Parasitol.

[ppat-0030192-b006] de Lara Capurro M, Coleman J, Beerntsen BT, Myles KM, Olson KE (2000). Virus-expressed, recombinant single-chain antibody blocks sporozoite infection of salivary glands in Plasmodium gallinaceum-infected Aedes aegypti. Am J Trop Med Hyg.

[ppat-0030192-b007] Ghosh AK, Ribolla PE, Jacobs-Lorena M (2001). Targeting *Plasmodium* ligands on mosquito salivary glands and midgut with a phage display peptide library. Proc Natl Acad Sci U S A.

[ppat-0030192-b008] Zieler H, Keister DB, Dvorak JA, Ribeiro JM (2001). A snake venom phospholipase A(2) blocks malaria parasite development in the mosquito midgut by inhibiting ookinete association with the midgut surface. J Exp Biol.

[ppat-0030192-b009] Rodriguez MC, Zamudio F, Torres JA, Gonzalez-Ceron L, Possani LD (1995). Effect of a cecropin-like synthetic peptide (Shiva-3) on the sporogonic development of Plasmodium berghei. Exp Parasitol.

[ppat-0030192-b010] Arrighi RB, Nakamura C, Miyake J, Hurd H, Burgess JG (2002). Design and activity of antimicrobial peptides against sporogonic-stage parasites causing murine malarias. Antimicrob Agents Chemother.

[ppat-0030192-b011] Nirmala X, James AA (2003). Engineering *Plasmodium*-refractory phenotypes in mosquitoes. Trends Parasitol.

[ppat-0030192-b012] Riehle MA, Srinivasan P, Moreira CK, Jacobs-Lorena M (2003). Towards genetic manipulation of wild mosquito populations to combat malaria: advances and challenges. J Exp Biol.

[ppat-0030192-b013] Ito J, Ghosh A, Moreira LA, Wimmer EA, Jacobs-Lorena M (2002). Transgenic anopheline mosquitoes impaired in transmission of a malaria parasite. Nature.

[ppat-0030192-b014] Moreira LA, Ito J, Ghosh A, Devenport M, Zieler H (2002). Bee venom phospholipase inhibits malaria parasite development in transgenic mosquitoes. J Biol Chem.

[ppat-0030192-b015] Moreira LA, Wang J, Collins FH, Jacobs-Lorena M (2004). Fitness of anopheline mosquitoes expressing transgenes that inhibit *Plasmodium* development. Genetics.

[ppat-0030192-b016] Ghosh A, Edwards MJ, Jacobs-Lorena M (2000). The journey of the malaria parasite in the mosquito: hopes for the new century. Parasitol Today.

[ppat-0030192-b017] Billker O, Lindo V, Panico M, Etienne AE, Paxton T (1998). Identification of xanthurenic acid as the putative inducer of malaria development in the mosquito. Nature.

[ppat-0030192-b018] Billker O, Dechamps S, Tewari R, Wenig G, Franke-Fayard B (2004). Calcium and a calcium-dependent protein kinase regulate gamete formation and mosquito transmission in a malaria parasite. Cell.

[ppat-0030192-b019] Hatakeyama T, Nagatomo H, Yamasaki N (1995). Interaction of the hemolytic lectin CEL-III from the marine invertebrate Cucumaria echinata with the erythrocyte membrane. J Biol Chem.

[ppat-0030192-b020] Oda T, Tsuru M, Hatakeyama T, Nagatomo H, Muramatsu T (1997). Temperature- and pH-dependent cytotoxic effect of the hemolytic lectin CEL-III from the marine invertebrate Cucumaria echinata on various cell lines. J Biochem (Tokyo).

[ppat-0030192-b021] Hatakeyama T, Miyamoto Y, Nagatomo H, Sallay I, Yamasaki N (1997). Carbohydrate-binding properties of the hemolytic lectin CEL-III from the holothuroidea Cucumaria echinata as analyzed using carbohydrate-coated microplate. J Biochem (Tokyo).

[ppat-0030192-b022] Hatakeyama T, Furukawa M, Nagatomo H, Yamasaki N, Mori T (1996). Oligomerization of the hemolytic lectin CEL-III from the marine invertebrate Cucumaria echinata induced by the binding of carbohydrate ligands. J Biol Chem.

[ppat-0030192-b023] Hatakeyama T, Suenaga T, Eto S, Niidome T, Aoyagi H (2004). Antibacterial activity of peptides derived from the C-terminal region of a hemolytic lectin, CEL-III, from the marine invertebrate Cucumaria echinata. J Biochem (Tokyo).

[ppat-0030192-b024] Hatakeyama T, Kohzaki H, Nagatomo H, Yamasaki N (1994). Purification and characterization of four Ca(2+)-dependent lectins from the marine invertebrate, Cucumaria echinata. J Biochem (Tokyo).

[ppat-0030192-b025] Edwards MJ, Lemos FJ, Donnelly-Doman M, Jacobs-Lorena M (1997). Rapid induction by a blood meal of a carboxypeptidase gene in the gut of the mosquito Anopheles gambiae. Insect Biochem Mol Biol.

[ppat-0030192-b026] Galun R, Koontz LC, Gwadz RW (1985). Engorgement response of anopheline mosquitoes to blood fractions and artificial solutions. Physiol Entomol.

[ppat-0030192-b027] Kouzuma Y, Suzuki Y, Nakano M, Matsuyama K, Tojo S (2003). Characterization of functional domains of the hemolytic lectin CEL-III from the marine invertebrate Cucumaria echinata. J Biochem (Tokyo).

[ppat-0030192-b028] Uchida T, Yamasaki T, Eto S, Sugawara H, Kurisu G (2004). Crystal structure of the hemolytic lectin CEL-III isolated from the marine invertebrate Cucumaria echinata: implications of domain structure for its membrane pore-formation mechanism. J Biol Chem.

[ppat-0030192-b029] Bremer E, van Dam G, Kroesen BJ, de Leij L, Helfrich W (2006). Targeted induction of apoptosis for cancer therapy: current progress and prospects. Trends Mol Med.

[ppat-0030192-b030] Vaughan JA, Noden BH, Beier JC (1994). Sporogonic development of cultured Plasmodium falciparum in six species of laboratory-reared Anopheles mosquitoes. Am J Trop Med Hyg.

[ppat-0030192-b031] Shahabuddin M, Fields I, Bulet P, Hoffmann JA, Miller LH (1998). Plasmodium gallinaceum: differential killing of some mosquito stages of the parasite by insect defensin. Exp Parasitol.

[ppat-0030192-b032] Curtis CF (1968). Possible use of translocations to fix desirable genes in insect pest populations. Nature.

[ppat-0030192-b033] Sinden RE, Smalley ME (1976). Gametocytes of Plasmodium falciparum: phagocytosis by leucocytes in vivo and in vitro. Trans R Soc Trop Med Hyg.

[ppat-0030192-b034] Lensen AH, Bolmer-Van de Vegte M, van Gemert GJ, Eling WM, Sauerwein RW (1997). Leukocytes in a Plasmodium falciparum-infected blood meal reduce transmission of malaria to *Anopheles mosquitoes*. Infect Immun.

[ppat-0030192-b035] Sinden RE, Crampton JM, Beard CB, Louis C (1997). Infection of mosquitoes with rodent malaria. Molecular biology of insect disease vectors: a methods manual.

[ppat-0030192-b036] Ponnudurai T, Lensen AH, Meis JF, Meuwissen JH (1986). Synchronization of Plasmodium falciparum gametocytes using an automated suspension culture system. Parasitology.

[ppat-0030192-b037] Ranawaka GR, Alejo-Blanco AR, Sinden RE (1994). Characterization of the effector mechanisms of a transmission-blocking antibody upon differentiation of Plasmodium berghei gametocytes into ookinetes in vitro. Parasitology.

[ppat-0030192-b038] Nakano M, Tabata S, Sugihara K, Kouzuma Y, Kimura M (1999). Primary structure of hemolytic lectin CEL-III from marine invertebrate Cucumaria echinata and its cDNA: structural similarity to the B-chain from plant lectin, ricin. Biochim Biophys Acta.

[ppat-0030192-b039] Skavdis G, Siden-Kiamos I, Muller HM, Crisanti A, Louis C (1996). Conserved function of Anopheles gambiae midgut-specific promoters in the fruitfly. EMBO J.

[ppat-0030192-b040] Yoshida S, Ioka D, Matsuoka H, Endo H, Ishii A (2001). Bacteria expressing single-chain immunotoxin inhibit malaria parasite development in mosquitoes. Mol Biochem Parasitol.

[ppat-0030192-b041] Walliker D, Quakyi IA, Wellems TE, McCutchan TF, Szarfman A (1987). Genetic analysis of the human malaria parasite Plasmodium falciparum. Science.

[ppat-0030192-b042] Lobo CA, Dhar R, Kumar N (1999). Immunization of mice with DNA-based Pfs25 elicits potent malaria transmission-blocking antibodies. Infect Immun.

[ppat-0030192-b043] Collins WE, Warren M, Skinner JC, Richardson BB, Kearse TS (1977). Infectivity of the Santa Lucia (El Salvador) strain of Plasmodium falciparum to different anophelines. J Parasitol.

