# Supplementary material for: Hemolytic C-Type Lectin CEL-III from Sea Cucumber Expressed in Transgenic Mosquitoes Impairs Malaria Parasite Development
Source: PLoS Pathog. 2007 Dec 21;3(12):e192. doi: 10.1371/journal.ppat.0030192 (PMC2151087; doi:10.1371/journal.ppat.0030192)
Supplement: Supplementary file 1 [file ppat.0030192.st001.doc]

**Supporting Information**

Table S1. Primer sequences

| Name | Sequence |
| --- | --- |
| pCEL3-F1 | CACCGAGCTCTGCAAGTTTTGTGCACGAATCCA |
| pCEL3-R1 | GCATGCTCACTCGAGAATGTCCGTGCAGAAAGTGCAAT |
| pAgCPA-F1 | TGGAGCAGGCGAGCGATAGTTTAATTTTCC |
| pAgCPA-R1 | ATTGCTCGTGCCCTGCTCGGCCCAGTGG |
| pAgCPA-F2 | AGGAGGCATGTCATAAGCCACTGA |
| pAgCPA-R2 | GCATGCTTTTTGGATCCGCCTCGGCCGCTTCGACACTGAG |
| pAgAntrp1-F1 | CTCAGTGTCGAAGCGGCCGAGGCGGATCCAAAAAGCATGCGATCTCGGCTTCAAAACGGTACTGGA |
| pAgAntrp1-R1 | GAGCTCGAATGGAACTGGCGGAAC |
| pCEL3-RT-F1 | GGTGTTTGACGATTGGGAGGT |
| pCEL3-R2 | AAGTGCAATCCTCGTTAGCACA |
| pAsCPA-F1 | ACGAAACGGCCCGGAAGGTGGGCGATCGGT |
| pAsCPA-R1 | AAGTCCCAGTTCCGGTTCGGATCAGCTCCA |
| pAgS7-F1 | GAAGGCGATCATCATCATCGTGCCG |
| pAgS7-R1 | CACGCATCGGTTTGGGCAGAATGCG |
